# Duplicate US1 Genes of Duck Enteritis Virus Encode a Non-essential Immediate Early Protein Localized to the Nucleus

**DOI:** 10.3389/fcimb.2019.00463

**Published:** 2020-01-17

**Authors:** Yangguang Li, Ying Wu, Mingshu Wang, YunChao Ma, Renyong Jia, Shun Chen, Dekang Zhu, Mafeng Liu, Qiao Yang, Xinxin Zhao, Shaqiu Zhang, Juan Huang, Xumin Ou, Sai Mao, Ling Zhang, Yunya Liu, Yanling Yu, Leichang Pan, Bin Tian, Mujeeb Ur Rehman, Xiaoyue Chen, Anchun Cheng

**Affiliations:** ^1^Institute of Preventive Veterinary Medicine, Sichuan Agricultural University, Wenjiang, China; ^2^Avian Disease Research Center, College of Veterinary Medicine of Sichuan Agricultural University, Wenjiang, China; ^3^Key Laboratory of Animal Disease and Human Health of Sichuan Province, Sichuan Agricultural University, Wenjiang, China

**Keywords:** herpesvirus, DEV, US1, ICP22, IE gene, non-essential, NLS

## Abstract

The duplicate US1 genes of duck enteritis virus (DEV) encode a protein with a conserved Herpes_IE68 domain, which was found to be closely related to the herpes virus immediate early regulatory protein family and is highly conserved among counterparts encoded by Herpes_IE68 genes. Previous studies found the homologous proteins HSV-1 ICP22 and VZV ORF63/ORF70 to be critical for virus transcription and replication. However, little is known about the DEV ICP22 protein. In this paper, we describe the characteristics of this protein based on pharmacological experiments, real-time quantitative Polymerase Chain Reaction, Western blot, and immunofluorescence assays. We also investigate the role of the protein in DEV replication via mutation of US1. As a result, we found that the DEV ICP22 protein is a non-essential immediate early protein predominantly located in the nucleus of infected DEF cells and that DEV replication is impaired by US1 deletion. We also found that ICP22 contains a classical nuclear localization signal (NLS) at 305-312AA, and ICP22 cannot enter the nucleus by itself after mutating residue 309.

## Introduction

Duck plague is an acute, febrile, septic, and lethal infectious disease of ducks, geese, and swans (Cheng, 2015). The causative agent of this virus is known as duck plague virus (DPV) or duck enteritis virus (DEV), belonging to the alpha-herpesvirus superfamily of Herpesviridae (Bukreyev et al., 2014). Similar to its homologs, the DEV genome consists of a linear, double-stranded DNA comprising a unique long (UL), a unique short (US), a unique short internal repeat (IRS), and a unique short terminal repeat (TRS) region (UL-IRS-US-TRS) (Wu et al., 2012). Traditionally, viral genes have been believed to be expressed in a temporal cascade sequentially composed of immediate early (IE), early (E), and late (L) genes during lytic infection (Alfonsodunn et al., 2017). IE genes are transcribed immediately upon infection without the requirement of *de novo* protein synthesis, and early genes are commonly used to regulate viral replication. Late proteins form the capsid or surface receptors.

Although some DEV genes have been studied in depth (Ming-Sheng et al., 2008, 2010; Hua et al., 2009, 2011; Chanjuan et al., 2010; Wei et al., 2010; Wang et al., 2011; Wu et al., 2011; Zhang et al., 2011, 2017; He et al., 2012, 2018; Ying et al., 2012; Liu et al., 2016; Gao et al., 2017; Liu C. et al., 2017; Liu T. et al., 2017; Feng et al., 2018; Ma et al., 2018; You et al., 2018; Zhao et al., 2019), information regarding the DEV US1 gene is extremely limited. It is known that the DEV US1 gene is 990 bp in length and duplicated within the inverted repeat sequences delineating the US region of the genome (Ying et al., 2012). The homolog of its encoded protein ICP22 has been well described in Herpes simplex virus types 1 and 2 (HSV-1 and HSV-2) (Barcy and Corey, 2001; Lei et al., 2012; Zaborowska et al., 2014), Pseudorabies virus (PRV) (Cai et al., 2016), Equine herpes virus types 1 and 4 (EHV-1 and EHV-4) (Holden et al., 1995; Kim et al., 1997; Meulen et al., 2006), Bovine herpes virus type 1 (BHV-1) (Köppel et al., 1997), and Varicella zoster virus (VZV) (Di et al., 2005; Ambagala and Cohen, 2007). As one of the most important immediate early protein of HSV-1, ICP22 plays an important role in virus replication and transcriptional regulation and is necessary for acute replication of HSV-1 in eyes and neurons as well as the establishment of HSV-1 latent infection (Fraser and Rice, 2005; Rice and Davido, 2013).

Shortly after HSV-1 enters susceptible cells, the viral genome is transported to the nucleus, after which HSV-1 effectively recruits the RNA Pol II transcription machinery of host cells to transcribe viral genes at a high level while inhibiting the transcription of most host genes. The mechanism by which Pol II preferentially transcribes viral genes over host genes has not been determined, but some physical changes occur in Pol II itself (Fraser and Rice, 2005). According to previous work, ICP22 mediates two completely different effects on Pol II: induction of Pol IIi formation and loss of Pol II ser-2 phosphorylation (Ser-2P) (Zaborowska et al., 2016). It has also been shown that ICP22 promotes recruitment of the viral genome by transcription elongation factors, such as the FACT complex, to facilitate the transcriptional expression of the viral L gene in the late stage of infection (Fox et al., 2017). Furthermore, in the lytic infection phase of HSV-1 infection, the nucleocapsid assembled in the nucleus needs to enter the cytoplasm after initial packaging in the perinuclear space (Newcomb et al., 2017), with ICP22 having a regulatory role; that is, initial effective packaging of the newly produced nucleocapsid of HSV-1 requires ICP22 (Yuhei et al., 2014). In addition, a novel function of ICP22 was recently identified, involving alteration of chaperone localization in host cells (Köppel et al., 1997). It can be seen from the above research that HSV-1 ICP22 regulates the transcriptional expression of certain viral genes to create a nuclear environment conducive to viral replication, thereby promoting effective virus replication in host cells. Therefore, ICP22 is of great significance to the life cycle of herpes virus in host cells as well as in the interaction between pathogens and host cells.

ORF63, the ICP22 homolog of VZV, which is critical for efficient establishment of latency (Ambagala and Cohen, 2007), does not affect RNAPII phosphorylation or host chaperones (Fraser and Rice, 2005). At the same time, other studies have reported that BICP22, the homolog of ICP22 in BHV-1, exerts a general repressive effect on each kinetic class (Köppel et al., 1997). This finding might indicate that ICP22 acts in a species- or genus-specific manner. At present, the properties of the duplicate DEV US1 genes and their encoded proteins have not been determined, and additional research is warranted to determine whether DEV ICP22 acts in a manner similar to its homologs.

To describe the DEV US1 gene and its encoded protein, an ICP22-specific antibody was prepared, after which RT-qPCR, WB and IFA were used to analyse the transcription, expression level and intracellular localization of the duplicated US1 gene of DEV after infection or eukaryotic transfection. The US1 gene type was assessed using the nucleic acid synthesis inhibitor ACV and the protein synthesis inhibitor CHX (Liu et al., 2015). The results showed that DEV ICP22 is an immediate early protein located in the nucleus of DEF cells, which contains a classical NLS at 305-314 AA of the ICP22 protein. To elucidate the roles of DEV US1 and its NLS in virus replication, a two-step homologous recombination technique was used for US1 mutating, the results showed the US1 gene and its 308-312AA is important for DEV replication. This is the first report of DEV US1 and these details regarding its role in virus replication will be helpful for further investigation of its function and mechanism.

## Materials and Methods

### Cells and Viruses

Monolayer cultures of duck embryo fibroblasts (DEFs) were propagated in Dulbecco's Modified Eagle Medium (DMEM; Gibco, USA) supplemented with 10% newborn bovine serum (NBS) and incubated at 37°C in a 5% CO2 humidified incubator. Wild-type DEV CHv (Ying et al., 2012) and its recombinant virus DEV BAC were generated in our laboratory (Ying et al., 2017).

### Plasmid Construction and Transfection

All enzymes used for cloning processes were purchased from Takara (Takara, China), except for MonClone™ Hi-Fusion Cloning Mix V2 (Monad, China). ICP22 ORFs (composed of 990 bp) were amplified via PCR from the genomic DNA of DEV using primers P1 and P2 (Table 1) and purified using a PCR gel purification kit (Tiangen, China). Subsequently, the products were inserted into correspondingly digested pEGFP-N1 and pet32a(+) vectors using EcoR I and Xho I QuickCut enzyme to create the recombinant plasmids EGFP-ICP22 and pet32a(+)-ICP22, and following the same steps for other plasmids. In this study, eukaryotic expression plasmids were transfected into 70–90% confluent cells using Lipofectamine™ 3000 Transfection Reagent (Invitrogen, USA) following the manufacturer's instructions.

**Table 1:** Primers used in this study.

**Primer**	**Sequence (5^**′**^-3^**′**^)**	**Purpose**	**Length (bp)**
P1F	CTACCGGACTCAGATCTCGAG ATGGCGACGGCA		
P1R	GTACCGTCGACTGCAGAATT CCACTCTTGGGGCGTTTT GTGGT	Eukaryotic expression of US1	990
P2F	CCATGGCTGATATCGGAT CCGAATTCTACAGAACTGCGA TAGAC	Prokaryotic expression of US1	990
P2R	CAGTGGTGGTGGTGGTGGTG CTCGAGACTCTTGGGGCGTTT TGTGGT		
P3F	CGTAGCGTCACATCAAGCAG	ICP22 quantitative primers	147
P3R	GCGTTTGGTCCCTATAACCTC		
P4F	TGGCATCCACGAAACTACC	β-Actin quantitative PCR primers	130
P4R	CTTCTGCATCCTGTCAGCGA		
P5F	CAATATATAAAAGGCTCTCGTT TACAGAACTGCGATAGACAGG ATGACGACGATAAGTAGGG	Replacement of the US1 gene by the kana-resistance cassette	1108
P5R	AGGTTAATACGCGCTTGCAGC ATATATCGCGACAGGTTTAGTC TATCGCAGTTCTGTAAACGAG AGCCTTTTATATATTGTTAACC AATTCTGATTAG		
P6F	ATGGCGACGGCATCGCG ACGGCCAAGCGGTCAAGCG TGCGAGGATGACGACGATAA GTAGGG	Replacement of the reverse US1 gene by the reverse kana-resistance cassette	1109
P6R	TTAACTCTTGGGGCGTTTTGTG GTACCCGCGAGTGCGCTCAC GCACGCTTGACCGCTTGGCC GTCGCGATGCCGTCGCCATTA ACCAATTCTGATTAG		
P7F	TCATTGCTCAATACGGGAAG	Identification of the US1 gene deletion	1500&510
P7R	GCGGTGTTTATTGACATCA		
P8F	GGACAGCGTACCACAGATAA	Amplification of the UL30 fragment	498
P8R	ACAAATCCCAAGCGTAG		
P9F	TTTTCCTCCTCCTCGCTGAGT	Probe primers	60
P9R	GGCCGGGTTTGCAGAAGT		
P10	FAM-CCCTGGGTACAAGCG-MGB	Probe	/
P11F	AAGTCCGGCCGGACTCAGATC TCGAGCATGAGCGAAAAATACATCG	Prokaryotic expression of GFPC2-β-Gal-US1/ΔNLS/NLS	3308
P11R	GATGCCGTCGCCATACTGCA GAATTCTTTTTGACACCAGACCAA		
P12R	TTGGTCCCTATCATACTGC AGAATTCTTTTTGACACCAG ACCAA		
P13F	ATAGAGGTTATAGGGACCGCA CGCAAACGGCCGACCACGAG GAGTATG	Prokaryotic expression of GFPC2-β-Gal-US1 amino acid mutation	2200
P13R	GTCGGCCGTTTGCGTGCGGT CCCTATAACCTCTATAACT		
P14F	AGGTTATAGGGACCAAAGCCA AACGGCCGACCACGAGGAGT ATG		
P14R	CGTGGTCGGCCGTTTGGCTTT GGTCCCTATAACCTCTAT		
P15F	TTATAGGGACCAAACGCGCAC GGCCGACCACGAGGAGTATG AGC		
P15R	TACTCCTCGTGGTCGGCCGTG CGCGTTTGGTCCCTATAACCT		
P16F	AGGGACCAAACGCAAAGCGC CGACCACGAGGAGTATGAGC GCA		
P16R	ATACTCCTCGTGGTCGGCGCT TTGCGTTTGGTCCCTATAA		
P17F	CAAACGCAAACGGGCGACC ACGAGGAGTATGAGCG CACTCG		
P17R	TCATACTCCTCGTGGTCGCCC GTTTGCGTTTGGTCCCTATAA		
P18F	GCTACCATTACCAGTTGGTCT GGTGTCAAAAAGAATTCTG		
P18R	AAAACGATTCCGAAGCCCAAC CTTTCATAGAAGGCG		
P19F	CGACTCTGAAAGCGAAGTTAT AGAGGTTATAGGGACCAAAGC CAGGATGACGACGATAAGTAG GG	Replacement of the US1 309aa by the kana-resistance cassette	1035
P19R	TACCCGCGAGTGCGCTCATAC TCCTCGTGGTCGGCCGTTTGG CTTTGGTCCCTATAACCTCTAT AACTTCGCTTTCAGAGTCGTTA ACCAATTCTGATTAG		
P20F	CTCCGACTCTGAAAGCGAAGT TATAGAGGTTATAGGGACCGC AGCCGCAGCGGCGACCACGA GGAGTATGAGCGCAGGATGA CGACGATAAGTAGGG	Replacement of the US1 308-312aa by the kana-resistance cassette	1035
P20R	GTTTTGTGGTACCCGCGAGTG CGCTCATACTCCTCGTGGTCG CCGCTGCGGCTGCGGTCCCT ATAACCTCTATAATTAACCAAT TCTGATTAG		

### Generation of a Polyclonal Antibody Against the Recombinant ICP22 Protein

As previously described Cheng et al. (2012), the plasmid pet32a(+)-ICP22 was transformed into *Escherichia coli* BL21 for protein expression. Bacterial pellets were resuspended in 20 mmol/L Tris-HCL buffer, and the supernatant was incubated with Ni-NTA his• Bind® Resin (Millipore, USA) at low temperature for 2 h. The samples were centrifuged to obtain a precipitate that was washed three times with washing solution to remove non-target proteins. The ICP22 protein was further purified by 10% SDS-PAGE gel and electroelution and used to generate a polyclonal antibody in mice.

### Real-Time Quantitative PCR (RT-qPCR)

RT-qPCR was performed primarily according to a previous method (He et al., 2018). Briefly, DEF cells were infected with DEV at an MOI of 10. Total RNA was isolated from the DEV-infected DEF cells at different time points post-infection (0.5, 2, 4, 8, 12, 24, 36, and 72 h) using TRIzol (Invitrogen, USA). cDNA was synthesized in a reverse transcriptase reaction using a PrimeScript™ RT reagent Kit with gDNA Eraser (Takara, China). Subsequently, qPCR was performed in a 10-μL reaction volume. The primers P3 and P4 used are listed in Table 1. The β-actin gene was used as an endogenous control to normalize differences in the amount of total RNA in each sample. RT-qPCR was performed in triplicate and analyzed using the 2^−Δ*CT*^ threshold cycle method.

### Pharmacological Inhibition Reaction

The procedure was performed as described previously (Liu et al., 2015). Briefly, the nucleic acid synthesis inhibitor ACV and the protein synthesis inhibitor CHX were used for determination of the US1 gene type. In brief, total RNA was isolated from DEV-infected DEF cells incubated with ACV or CHX at 24 h post-infection and reverse-transcribed into cDNA. The cDNA was used for PCR analysis, and the product was identified using agarose gel electrophoresis.

### Western Blotting Analysis

As previously described Li et al. (2011), DEFs infected with DEV or treated with drugs were harvested at the indicated time points for sample preparation. The samples were separated by 10% SDS-PAGE and transferred to polyvinylidene fluoride (Millipore, USA) membranes. The membranes were blocked with 5% BSA TBS buffer containing 0.05% Tween-20 for 2 h and incubated with the diluted primary antibody for 2 h at 37°C. The membrane was washed with TBST and incubated with goat anti-mouse HRP-labeled IgG (Abcam, Britain) secondary antibodies for 1 h at 37°C. After washing, the membrane was developed using an ECL kit.

### Immunofluorescence Assay

IFA was performed as previously described Liu et al. (2015), with minor modifications. Briefly, 4% paraformaldehyde was used to fix DEF cells grown on coverslips in 6-well plates at different time points. The cells were permeabilized in 0.25% Triton X-100 and blocked in 5% BSA at 37°C. The anti-ICP22 antibody and FITC/TRITC-conjugated IgG (Invitrogen, USA) were used as primary and secondary antibodies, respectively. All antibodies were diluted in 1% BSA PBS. The cells were treated with 4'6-diamidino-2-phenylindole (DAPI) for 15 min to stain the nucleus. Images were captured using a fluorescence microscope after the coverslips were sealed onto glass slides with glycerine buffer.

### Construction and Identification of the US1 Mutant Viruses

The Red-mediated recombination scheme is shown in **Figure 5A** (consider the first US1 gene deletion as an example), primarily referring to the method of Tischer et al. (2006, 2010). Transfection of DEV in BAC-ΔUS1-GS1783 and DEV in BAC-2ΔUS1-GS1783 plasmids into DEFs was subsequently performed; when fluorescence was observed, the cell medium was collected to obtain recombinant virus DEV CHv-BAC-G-ΔUS1 (BAC-ΔUS1) and DEV CHv-BAC-G-2ΔUS1 (BAC-2ΔUS1) (**Figure 5B**). The identification of BAC-ΔUS1 and BAC-2ΔUS1 is depicted in **Figure 6**. As shown in **Figure 6A**, 1 μg of plasmid was digested for 30 min with *BamH* I or *Xho* I at 37°C, and then agarose gel electrophoresis was carried out at 30 V for ~10 h. We used P7 to identify recombinant viral genomic DNA to ensure that the recombinant virus was not contaminated by the parental strain BAC (**Figure 6B**). The construction process of DEV CHv-BAC-G-ΔUS1-R309A (BAC-ΔUS1-R309A) and DEV CHv-BAC-G-ΔUS1-308-312AA (BAC-ΔUS1-308-312AA) is the same as above.

### Growth Curve and Determination of DNA Copies

Growth curve analysis was performed as described previously (Ma et al., 2018). Briefly, the growth kinetics of US1 mutant viruses were compared to that of the parental strain. Cell cultures were infected at an MOI of 0.01 (multi-step assay) or 2 (single-step assay). After 2 h of adsorption, the cells were washed and then overlaid with DMEM containing 2% NBS. Supernatants and infected cells were separately harvested at 24, 48, 72, 96 h (multi-step assay) or 6, 12, 18, 24, 36 h (single-step assay) at successive intervals, and the amount of infectious virus was determined by plaque assay using DEF cells.

The viral dosage and sampling time points were the same as for the growth curve detection described above. First, the UL30 DNA fragment was amplified with primer P8, and the fragment diluted in a 10-fold gradient was used as a template followed by the addition of primer P9 and probe P10 for qPCR to construct a standard curve: Y = −4.262X+43.675. Primers P8, 9, and 10 used in this part are listed in Table 1. The viral DNA in the samples was extracted using a Viral RNA/DNA Extraction Kit (Takara, China). qPCR was performed after the addition of primer P9 and probe P10, and viral DNA copies were estimated at various time points using the standard curve. All of the analyses were performed independently in triplicate with the standard error.

## Results

### Generation of a Polyclonal Antibody Against the Recombinant ICP22 Protein

**Figure 1:**
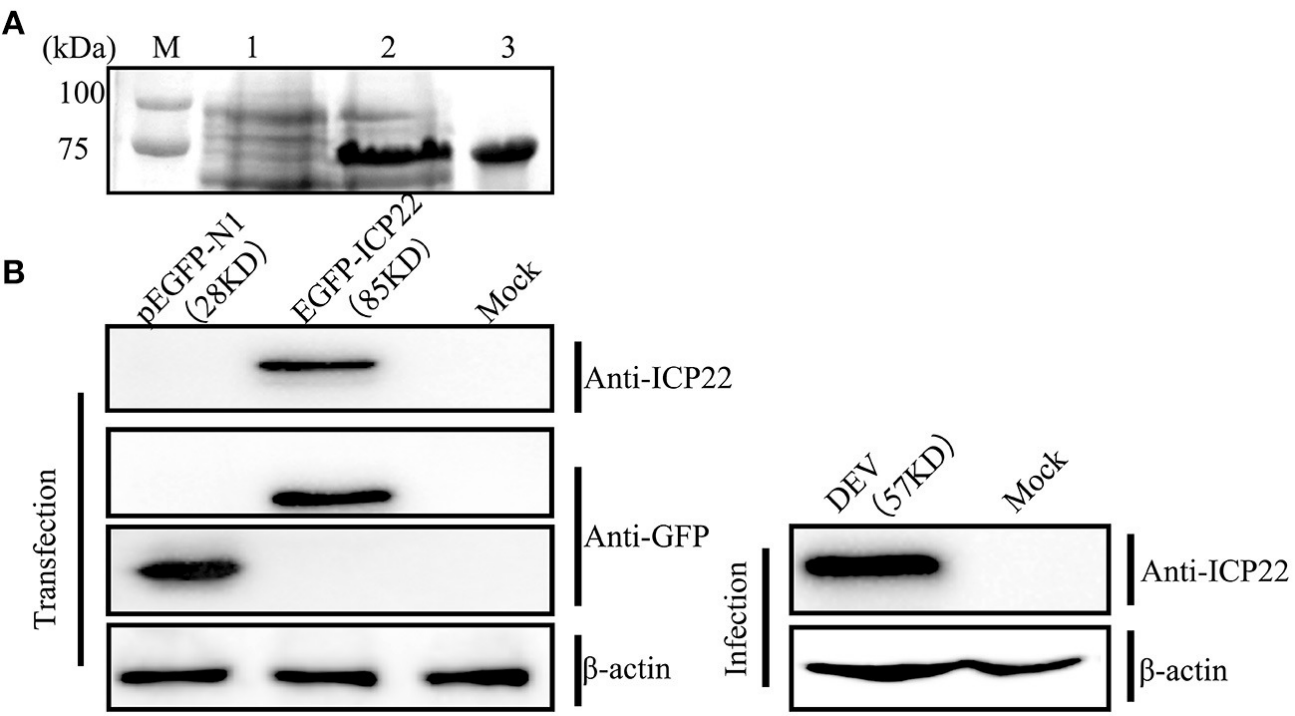
Prokaryotic expression of DEV ICP22 and identification of the ICP22 protein. **(A)** M, Protein molecular mass marker; Lane 1, untreated samples; lane 2, recombinant bacterial supernatant after IPTG induction; lane 3, the purified ICP22 protein. **(B)** Identification of the ICP22 protein by the prepared anti-ICP22 antibody in the eukaryotic expression plasmid EGFP-ICP22 transfection group (left panel) or DEV infection group (right panel).

Polyclonal antiserum was generated using purified protein ICP22 to evaluate changes in intracellular localization and protein expression of DEV US1. As shown in Figure 1A, a distinct band with a molecular mass of ~75 kDa was visible when pet32a(+)-ICP22 expression was induced using IPTG in *E. coli* BL21 at 37°C for 6 h; the ICP22 protein molecular mass is ~35 kDa, that of the His-tagged protein is approximately 17 kDa, and pet32a(+) is approximately 20 kDa. Next, the fusion protein was purified by gel electrophoresis and electroelution to generate the mouse anti-ICP22 protein polyclonal antibody. After a total of 5 weeks, polyclonal sera were collected, and the ELISA titer of the anti-US1 antibody was determined to be 1:32,000.

DEV was individually inoculated or transfected into DEFs with eukaryotic expression plasmid EGFP-ICP22 and tested against each other using the ICP22 antibody and a commercial GFP antibody to assess the specificity of the homemade antibody. As shown in Figure 1B, the EGFP-ICP22 fusion protein is ~85 kDa (left panel), whereas an ~57-kDa band was observed after DEV infection (right panel).

### Transcription Kinetics of the DEV US1 Gene

**Figure 2:**
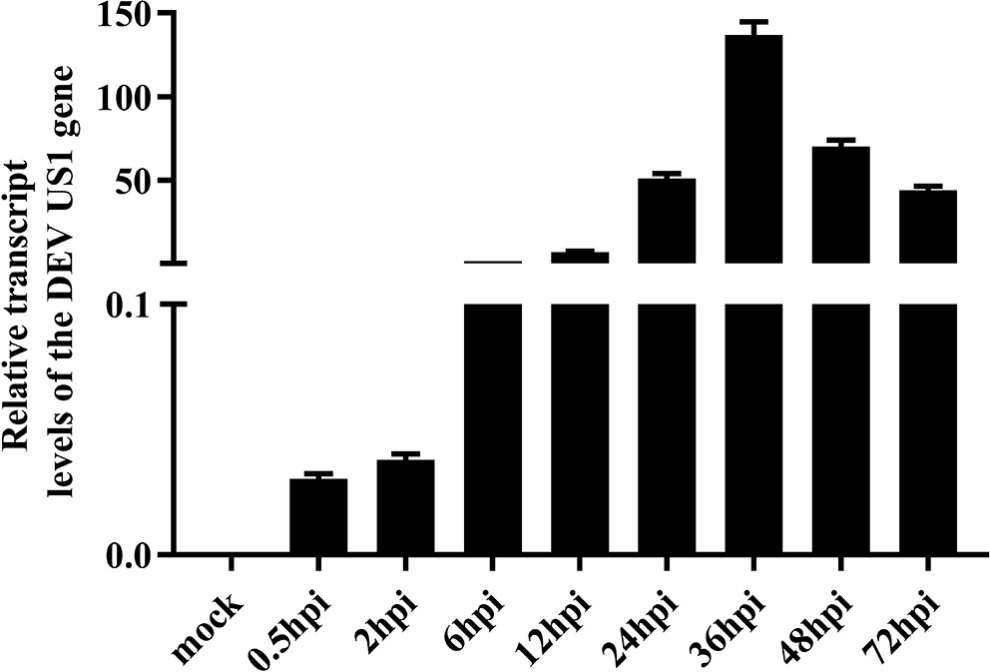
Transcriptional analysis of the DEV US1 gene in infected cells with 10 MOI DEV; the average relative content of the DEV US1 gene transcripts was calculated at the indicated time points using GraphPad Prism 8.0 software. Each time point was examined in triplicate with the standard error.

The transcription kinetics of the DEV US1 gene were plotted to investigate variations in the transcriptional level of the DEV US1 gene in DEFs during infection. The results of RT-qPCR showed that the US1 gene began to be transcribed at 0.5 h after infection, with the amount of transcription increasing with time and peaking at 36 h after infection, similar to the transcriptional profile of the immediate early gene UL54 of DEV (Liu et al., 2015). After reaching the peak, the relative transcription level began to decrease gradually (Figure 2).

### Dynamic Expression of the DEV US1-Encoded Protein in DEFs

**Figure 3:**
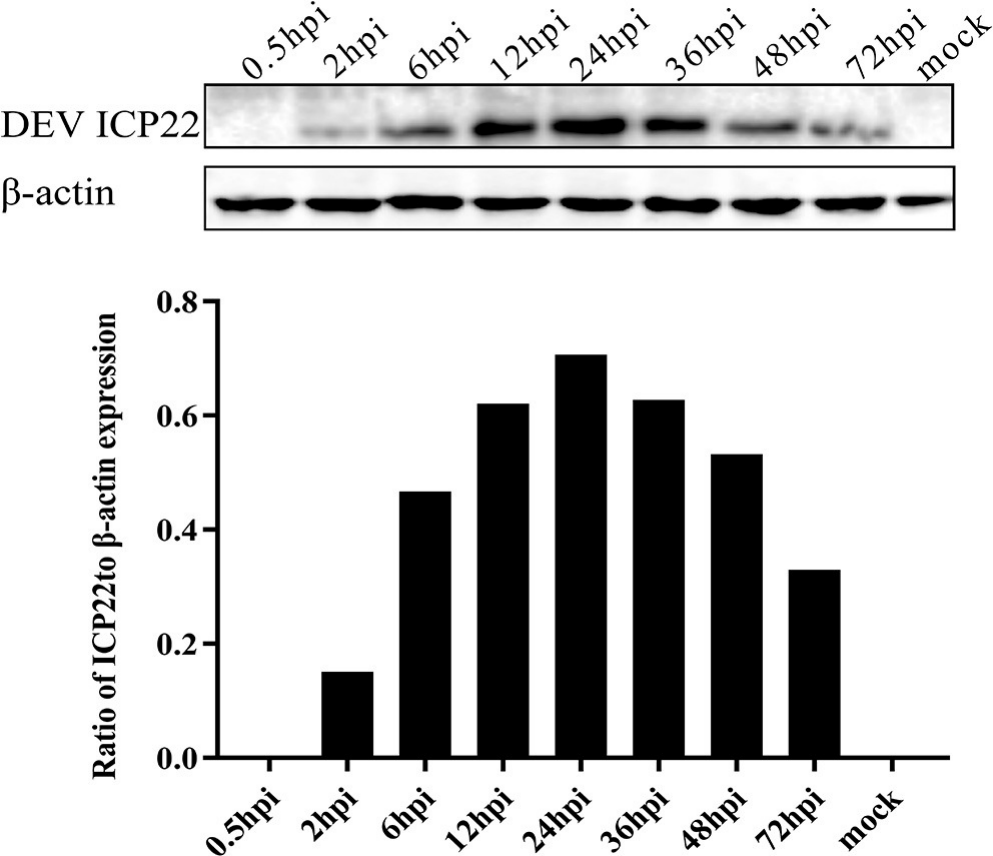
Dynamic expression of ICP22 in DEV-infected cells. Expression of ICP22 in DEFs with 10 MOI DEV was examined by Western blot assay (top panel). The quantitative result of WB is shown in the bottom panel. Mock-infected DEFs were used as a control.

To analyse the dynamic expression level of the ICP22 protein in infected cells more intuitively, samples were lysed at different times after viral infection for Western blot analysis. The US1-encoded ICP22 protein could be detected at 2 h after virus infection, and its expression level was stable from 12 to 36 hpi, peaked at 24 hpi, and decreased significantly after 48 hpi. Expression of the endo-reference protein β-actin was stable throughout infection (Figure 3).

### Pharmacological Inhibition Test

**Figure 4:**
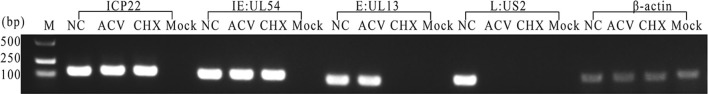
Identification of the US1 gene type in DEV-infected cells. M, DL2000 Marker; ACV group, DEV-infected cells treated with 300 μg/mL nucleic acid synthesis inhibitor ACV; CHX group, DEV-infected cells treated with 50 μg/mL the protein synthesis inhibitor CHX; NC group, DEV-infected cells without drugs; Mock group, mock-infected DEFs were used as a control.

Drug inhibition experiments were performed to determine US1 gene types. As shown in Figure 4, DEV US1 was still detected after treatment with the nucleic acid synthesis inhibitor ACV or the protein synthesis inhibitor CHX, which was the same as that of the measured immediate early gene UL54 (Liu et al., 2015). The reported E gene UL13 (Hu et al., 2017), L gene US2 (Gao et al., 2015), and β-actin were also detected as controls. The positive and negative lanes represent uninfected and infected DEF cells without any treatment, respectively. The pharmacological inhibition test indicated that the DEV US1 gene is an immediate early gene.

### Construction and Identification of the Duplicated US1 Deletion Mutant

**Figure 5:**
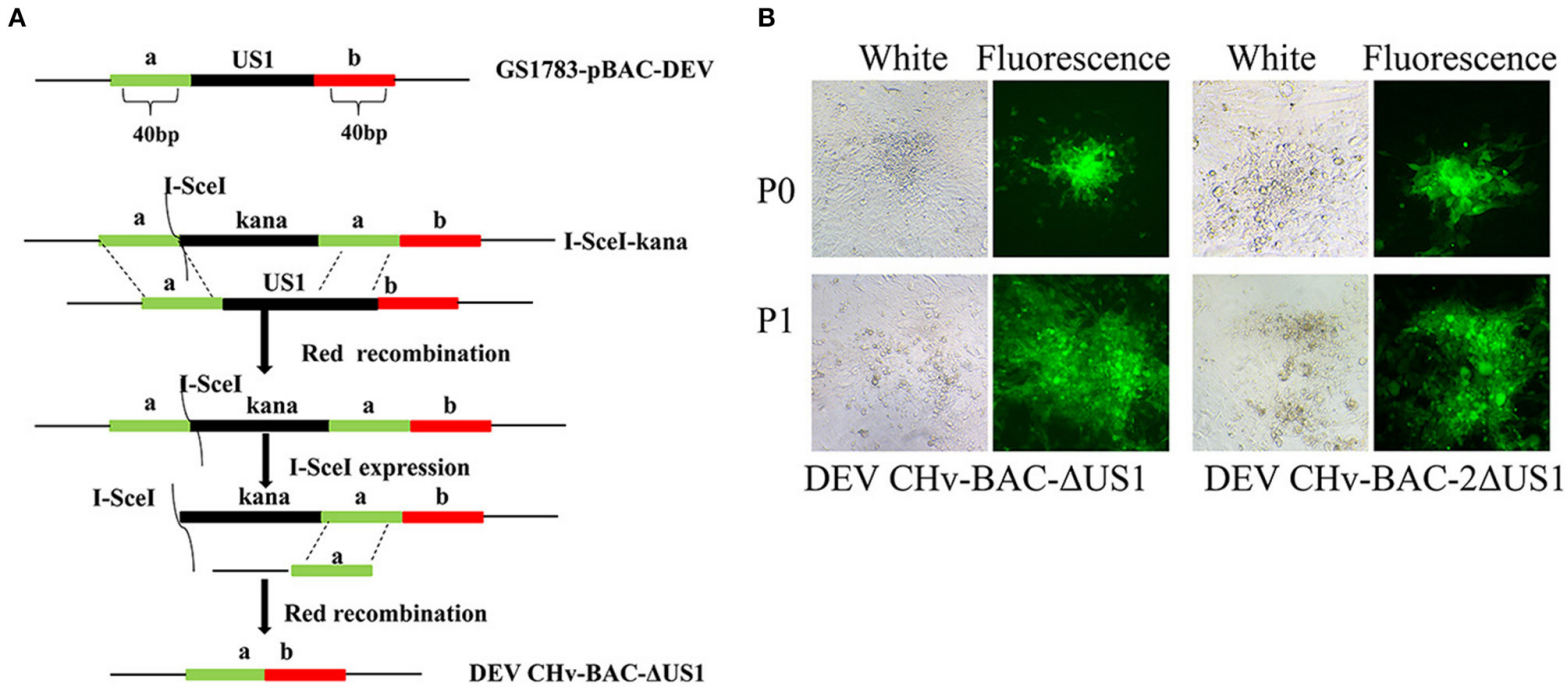
Construction of DEV CHv-BAC-ΔUS1 and DEV CHv-BAC-2ΔUS1. **(A)** Schematic diagram of constructing the duplicated US1 deletion mutant using the Red recombinant system. **(B)** DEV CHv-BAC-ΔUS1 and DEV CHv-BAC-2ΔUS1 were rescued in DEF cells.

The construction of recombinant DEV in BAC-ΔUS1-GS1783 and DEV in BAC-2ΔUS1-GS1783 were carried out following the flow diagram displayed in Figure 5A. As shown in Figure 5B, plasmids from the positive clone were transfected into DEFs. After fluorescent spots appeared, the cell medium was collected, and the recombinant viruses DEV CHv-BAC-ΔUS1 and DEV CHv-BAC-2ΔUS1 with deletion of the US1 gene were obtained.

**Figure 6:**
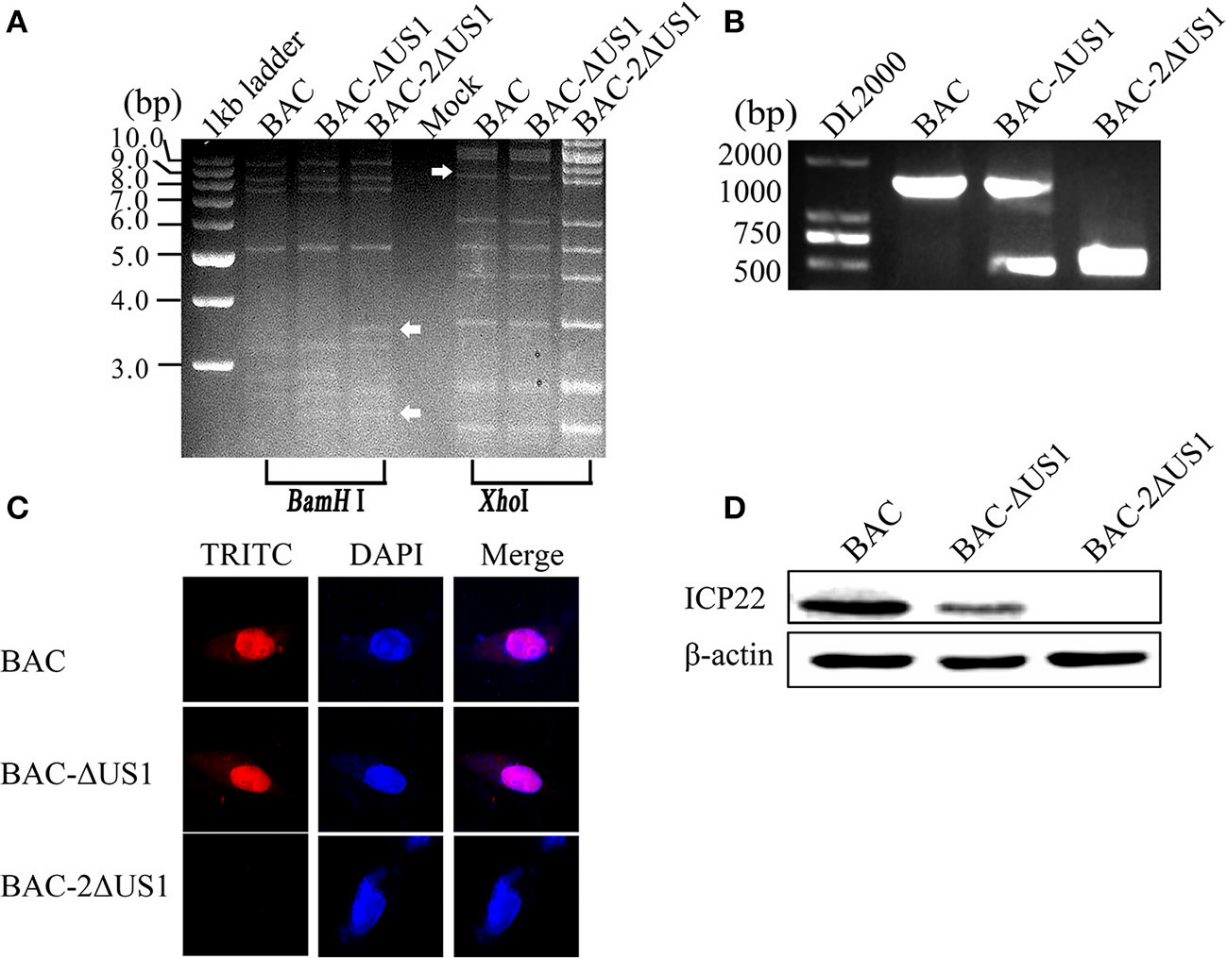
Identification of BAC-ΔUS1 and BAC-2ΔUS1. **(A)** Identification of BAC-ΔUS1 and BAC-2ΔUS1 by enzyme digestion. M, 1 kb ladder marker; lane 4, negative control. **(B)** Identification of DEV-ΔUS1 and DEV-2ΔUS1 by PCR. **(C)** Identification of BAC-ΔUS1 and BAC-2ΔUS1 by IFA. **(D)** Identification of BAC-ΔUS1 and BAC-2ΔUS1 by Western blot analysis.

Identification of recombinant virus DEV CHv-BAC-ΔUS1 (BAC-ΔUS1) and DEV CHv-BAC-2ΔUS1 (BAC-2ΔUS1) were carried out by enzyme digestion, IFA and Western blotting. As shown in Figure 6A, the DEV CHv-BAC and BAC-ΔUS1, 2ΔUS1 plasmids were digested by *BamH* I or *Xho* I into multiple fragments of different sizes, and the band differences are indicated by arrows. In IFA and Western blot analyses, no specific red fluorescence or specific band was observed for DEV-2ΔUS1, which indicated that the DEV US1 deletion (BAC-ΔUS1 and BAC-2ΔUS1) were successfully constructed (Figures 6C,D).

### Determination of Growth and DNA Copies of US1 Mutants

**Figure 7:**
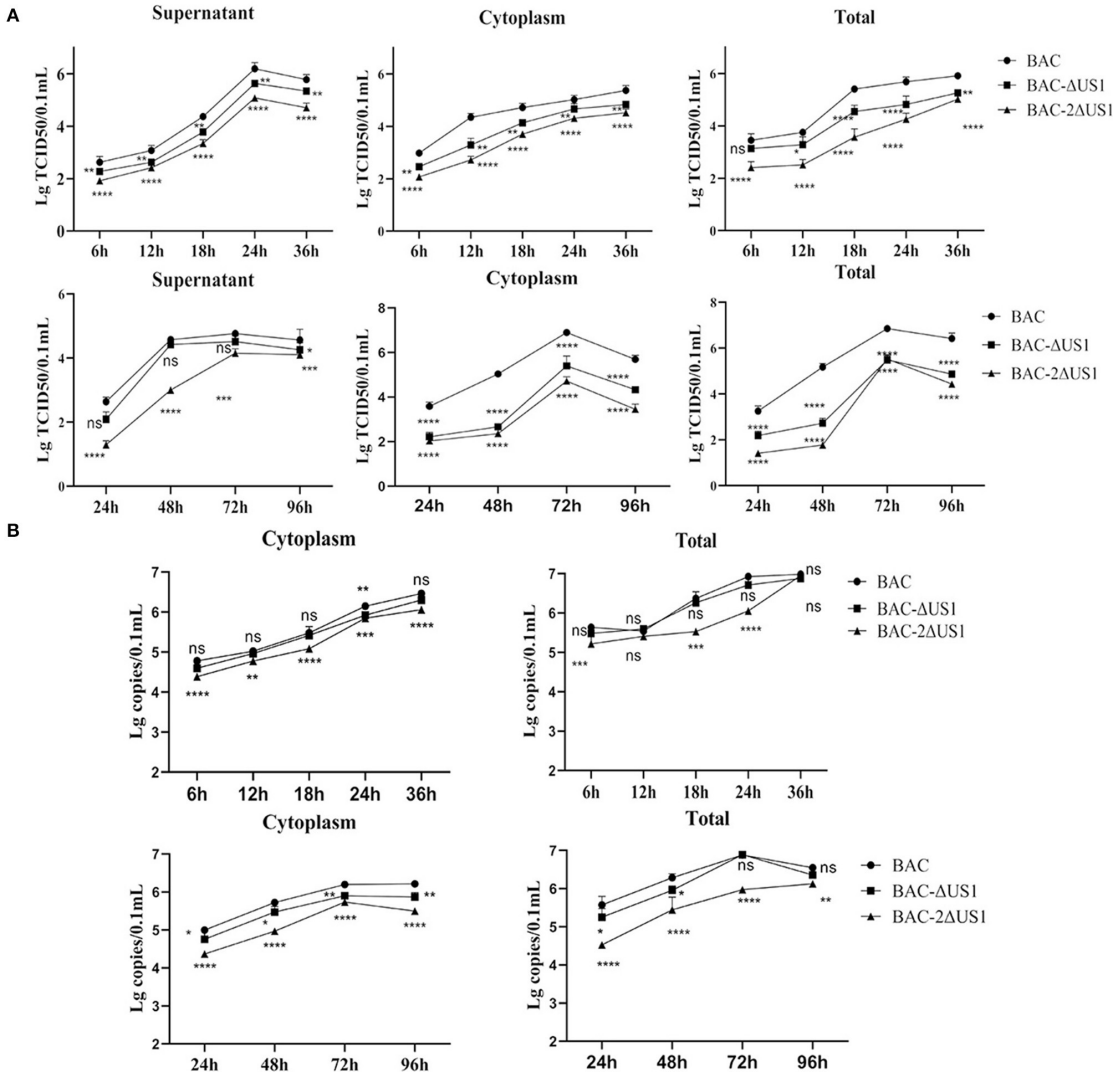
Viral titres and viral copies of one-step and multi-step replication kinetics. **(A)** Viral titer in the cytoplasm, supernatant and total of one-step growth assays (top panel) and multi-step replication kinetics (bottom panel). **(B)** Viral copies in the cytoplasm and total of one-step growth assays (top panel) and multi-step replication kinetics (bottom panel). Each time point was measured in triplicate, with the standard error indicated. A representative experiment with at least three repeats was performed. Ns represents no significance, *p < 0.1, **p < 0.01, ***p < 0.001 and ****p < 0.0001.

To better characterize ICP22, we evaluated the role of US1 in DEV infection *in vitro* by one-step and multi-step growth assays with DEF cells (Figure 7A). The results showed that after infection of three strains in DEF cells at the same multiplicity of infection, a similar proliferation pattern was observed, with severe replication defects after deletion of US1. The virus titer of the deletion strain was significantly lower than that of the parent strain; in particular, at 18 h (2 MOI) and 48 h (0.01 MOI) after infection, 2ΔUS1 was nearly 100 times lower than DEV BAC. To further analyse the role of the US1 gene in viral replication, we detected the number of viral copies in the samples. Compared with BAC, a notable decrease was observed for -BAC-2ΔUS1 (Figure 7B), coinciding with the results of the growth curves.

### Intracellular Localization of ICP22

**Figure 8:**
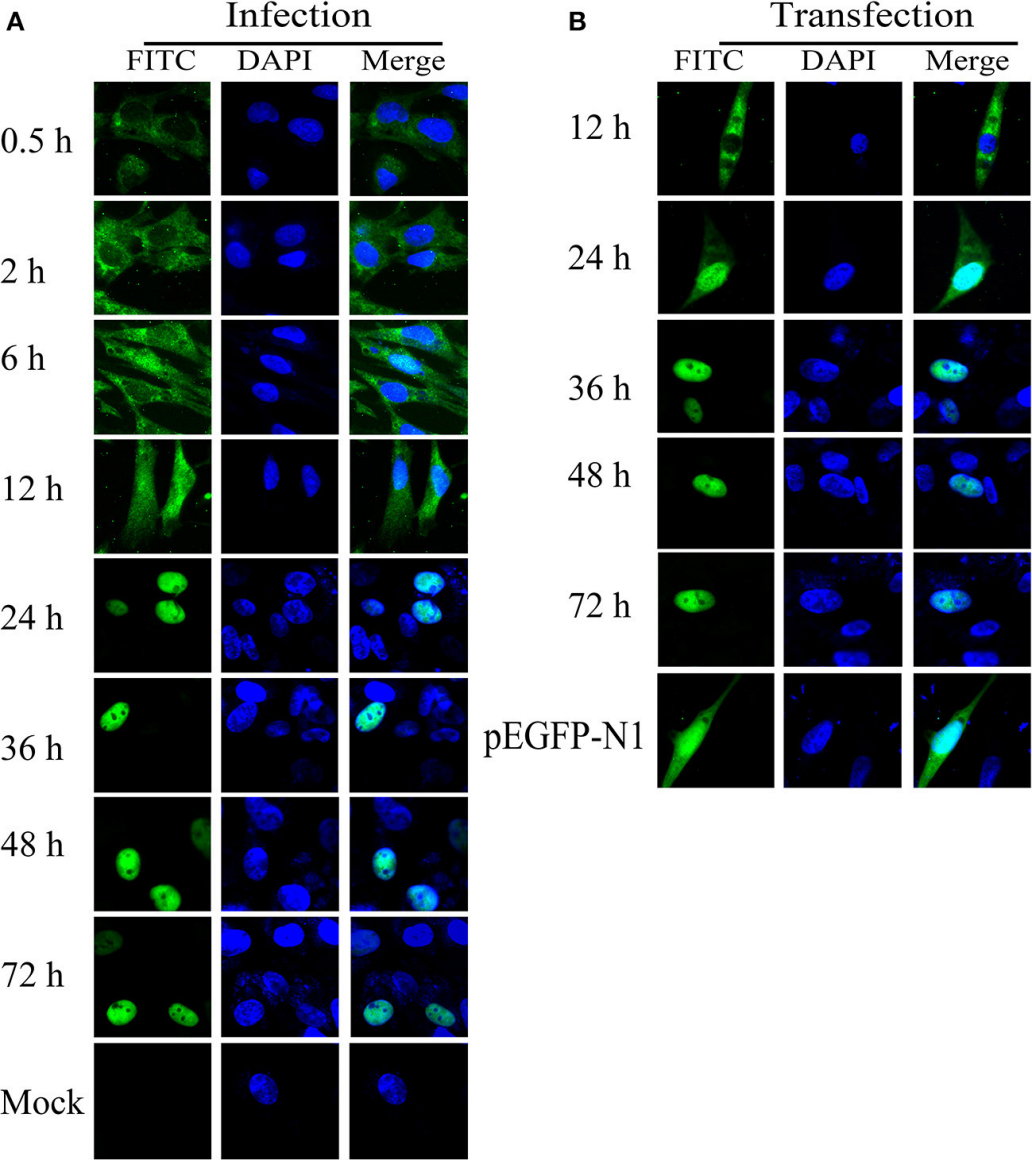
Intracellular localization of ICP22 in DEFs. **(A)** Intracellular localization of ICP22 in infected DEFs at different time points with 10 MOI; mock was used as a control. **(B)** Intracellular localization of ICP22 at different time points in EGFP-ICP22-transfected DEFs. pEGFP-N1 was used as a control.

The intracellular distribution of ICP22 was confirmed through IFA using mouse anti-ICP22 serum and FITC-conjugated goat anti-mouse IgG. As shown in Figure 8A, IFA results indicated that ICP22 began to enter the nucleus at 6 h after DEV infection, and all ICP22 had entered the nucleus at 24 h. To determine whether ICP22 enters the nucleus by itself or with other viral proteins, EGFP-ICP22 was transfected into DEFs for analysis of ICP22 distribution. We found that ICP22 began to enter the nucleus at 12 h after transfection, with all inside the nucleus at 36 h (Figure 8B).

### ICP22 Is Located in Nucleus Independent of NLS Motif in Infected DEFs

**Figure 9:**
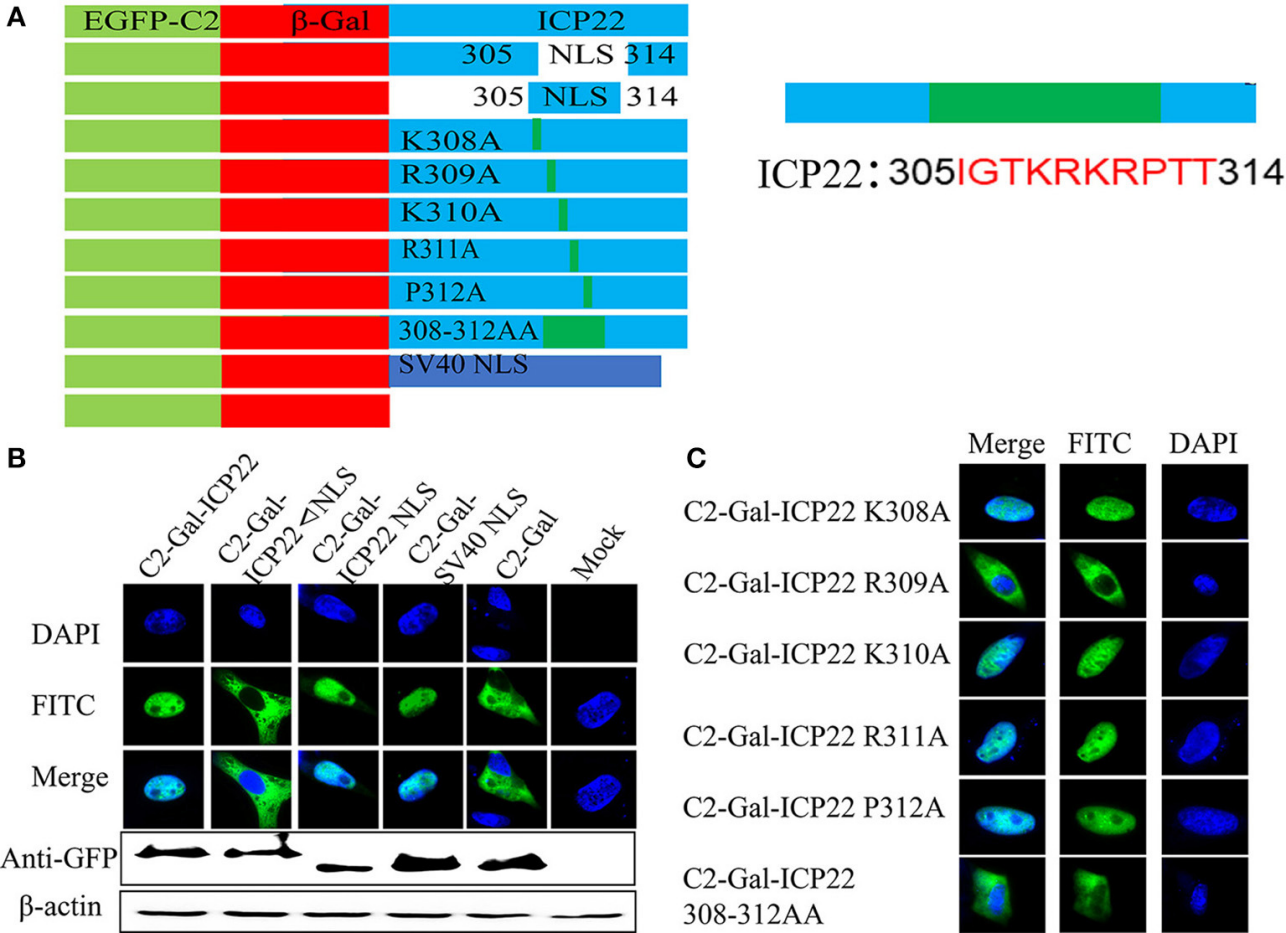
Screening for key amino acids in NLS after transfection. **(A)** Schematic diagram of the ICP22 plasmids. **(B)** Localization of ICP22 in different plasmids transfected DEFs. mock was used as a control. **(C)** Localization of ICP22 in different mutation plasmids transfected DEFs.

A series of plasmids were constructed to investigate how does ICP22 get into the nucleus (Figure 9A). As shown in Figure 9B, C2-β-Gal-ICP22 and C2-β-Gal-ICP22 NLS were localized to the nucleus, which was consistent with the positive control group C2-SV40 NLS-β-Gal. However, the NLS deleted plasmid C2-β-Gal-ICP22 ΔNLS was localized in the cytoplasm, which was consisting with that of C2-β-Gal (Chen et al., 2018). Further mutating of alkaline acid K/R in 308-314AA to alanine found the C2-β-Gal-ICP22 R309A and C2-β-Gal-ICP22 308-314AA were localized in the cytoplasm consisting with C2-β-Gal.

**Figure 10:**
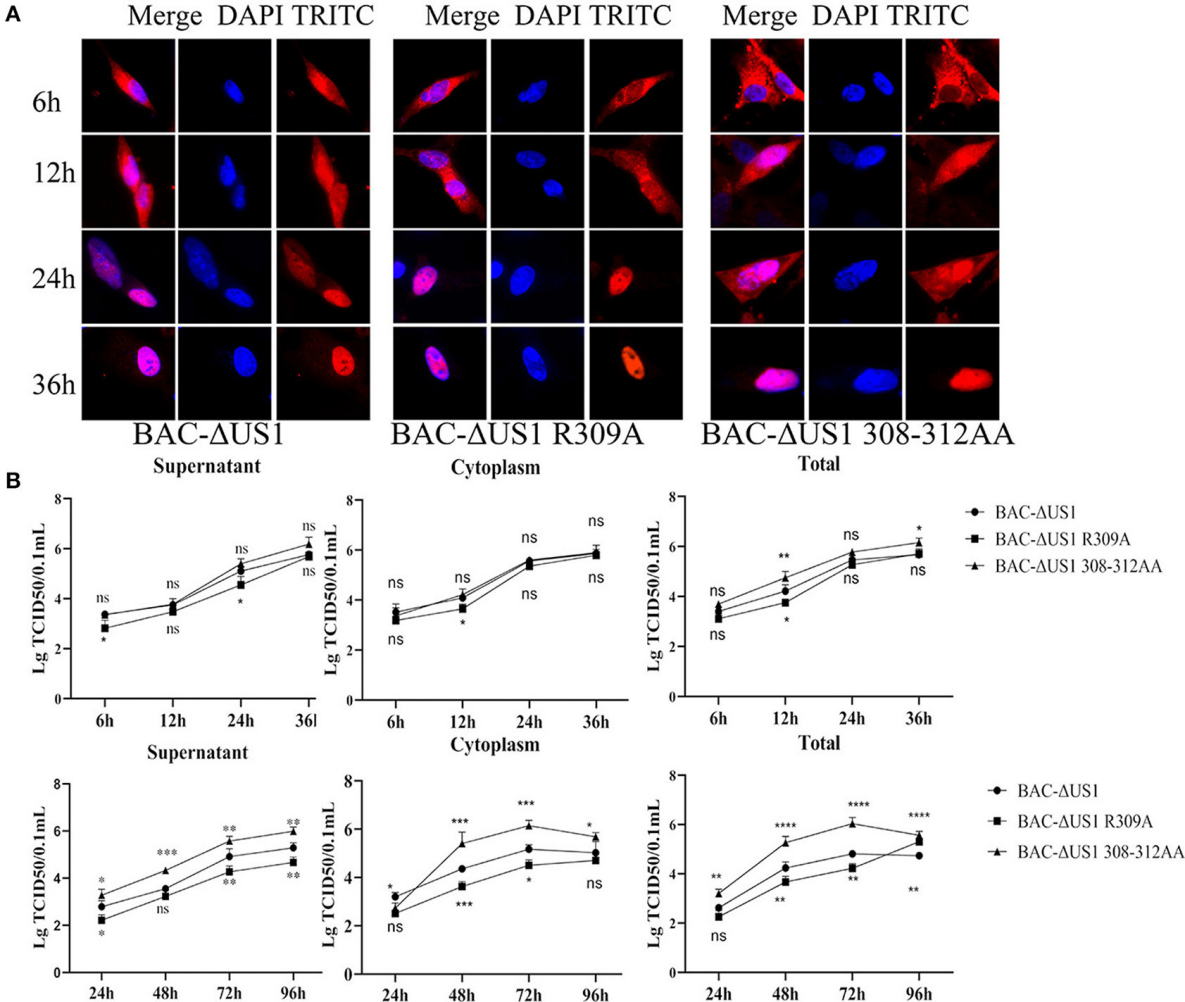
Intracellular localization of ICP22 and viral titres of one-step and multi-step replication kinetics. **(A)** Intracellular localization of ICP22 in infected DEFs at different time points with 10 MOI BAC-ΔUS1, BAC-ΔUS1 R309A, and BAC-ΔUS1 308-312AA. **(B)** Viral titer in the cytoplasm, supernatant, and total of one-step growth assays (top panel) and multi-step replication kinetics (bottom panel). Each time point was measured in triplicate, with the standard error indicated. A representative experiment with at least three repeats was performed. Ns represents no significance, *p < 0.1, **p < 0.01, ***p < 0.001 and ****p < 0.0001.

Based on the above results, point mutation viruses DEV CHV-BAC-ΔUS1 R309A and 308-312AA were constructed using Red recombinant system. As shown in Figure 10A, although ICP22 began to enter the nucleus at different time points after different virus infection, it could be located in the nucleus eventually. To better characterize the effect of DEV ICP22 R309A and 308-312AA on virus titer, we carried out the one-step and multi-step growth assays *in vitro* (Figure 10B). The result showed differential performance: the virus titer of the 308-312AA mutant was significantly higher than parent strain BAC-ΔUS1, even nearly 100 times higher than DEV BAC-ΔUS1 at 48 and 72 h after infection, but there is no obvious significant difference between the proliferation pattern between BAC-R309A and BAC-ΔUS10 (0.01MOI) and one-step growth assays.

## Discussion

To prepare the anti-ICP22 polyclonal antibody, the recombinant expression plasmid pet32a(+)-ICP22 was constructed. To test the specificity of the homemade antibody, DEFs were transfected with EGFP-ICP22 or infection with DEV, demonstrating that the prepared ICP22 antibody can recognize ICP22 specifically in transfection or infection assays and that ICP22 expressed in DEFs is ~57 kDa. Further commercial GFP antibody detection confirmed our findings. However, in our previous prokaryotic expression analysis, the ICP22 protein was found to be ~35 kDa. These results suggest that some modifications occurred during eukaryotic expression, resulting in a migration of ~22 kDa. It has been reported that modification of ICP22 is of great significance for its functions and occurs in other viruses; among them, different degrees of modification have been widely reported (Poon et al., 2000; O'Toole et al., 2003; Hoover et al., 2006; Bastian and Rice, 2009). According to bioinformatics analysis (Figure S1), DEV ICP22 has multiple phosphorylation and glycosylation sites, which may be related to the observed migration pattern. Further research will focus on ICP22 modifications and effects on its function.

As mentioned earlier, lytic herpesvirus genes are believed to be expressed in a cascade sequentially composed of IE, E, and L genes during lytic infection (Alfonsodunn et al., 2017). We performed the following analyses to identify the US1 gene type. First, we investigated the relative expression of the DEV US1 gene in DEV-infected DEF cells at different time points using RT-qPCR. The results showed transcript expression as early as 0.5 h that gradually increased until peaking at 24 h (Figure 2). Subsequently, we detected the dynamic expression of ICP22 at the protein level at specific time points and transcript levels. The ICP22 protein was detected at 2 h after infection, and its expression level peaked at 24 h, confirming the results of the translation studies and indicating that ICP22 can be expressed very early after infection with DEV. Finally, we identified the gene type of US1 using the nucleic acid synthesis inhibitor ACV and the protein synthesis inhibitor CHX (Liu et al., 2015). The pharmacological inhibition test showed that US1 was not affected by these two drugs. Combined with the above three tests, it can be determined that US1 is an immediate early gene, as is the reference gene UL54 (Liu et al., 2015).

US1 deletion mutants were also generated to investigate the roles of ICP22 in virus replication. The successful rescue of DEV CHv-BAC-ΔUS1 and DEV CHv-BAC-2ΔUS1 recombinant strains indicated that the US1 gene is not required for DEV replication. The ensuing examination of the growth kinetics and viral DNA copies of US1 deletion mutants and parental virus showed a lower viral titer and DNA copy number for DEV-CHv-BAC-2ΔUS1, indicating severe replication defects after deletion of the duplicate US1 genes, further suggesting that the US1 gene is important for DEV replication.

The intracellular localization of the protein is the basis for understanding the functions of a protein. In recent years, there have been reports that the ICP22 encoded by HSV-1 localizes to the cell nucleus independently of other viral proteins (Stelz et al., 2002). HSV-1 infection greatly alters the location of many host chaperones, including Hsc70, Hsp70, Hsp40, and Hsp90, causing their accumulation in the nucleosome, a region known as the virus-induced chaperone-enriched domain (VICE). Other studies have also reported that ICP22 localizes to VICE domains and is required for VICE domain formation during productive viral infection (Bastian et al., 2010). In our study, we found that ICP22 began to enter the nucleus at 6 h after DEV infection and that all of the protein had entered the nucleus at 24 h after DEV infection. To explore whether ICP22 localizes via active or passive transport, we constructed the ICP22 eukaryotic expression plasmid EGFP-ICP22 for transfection. As illustrated in Figure 1, the molecular weight of EGFP-ICP22 is ~85 kDa, which is considerably <50 kDa (Macara, 2001; Sorokin et al., 2007). EGFP-ICP22 began to enter the nucleus at 12 h after transfection, and all had entered the nucleus at 36 h, suggesting that the ICP22 protein has the ability to actively enter the nucleus. A series of plasmids and viruses were constructed to investigate how does ICP22 get into the nucleus. As shown in Figures 9, 10, we can conclude that the predicted NLS motif has strong nuclear import ability similar to that of SV40-NLS, and residue 309 is the key amino acid for determination of DEV ICP22 localization after transfection. But the NLS motif is not necessary for the localization of ICP22 in infected DEFs, and the delay of entering nucleus associated with DEV growth *in vitro*. According the previous studies, neither ICP4 nor ICP0 were recruited into NPDs (Newly synthesized Protein Domains, NPDs), while early in infection ICP22 was selectively recruited (Teo et al., 2016). And the ICP27 interacts with the C-terminal domain and is involved in the recruitment of RNAP II to viral transcription and replication compartments. During infection with ICP27 mutants that are unable to recruit RNAP II to viral replication sites, viral transcript levels were greatly reduced, viral replication compartments were poorly formed and Hsc70 focus formation was curtailed (Li et al., 2008). Combining the interaction between ICP22 and RNAP II, we speculate that ICP27 cooperates with the host protein to transport ICP22 to the nucleus. In addition, it is also possible that ICP22 contains other weak NLS. Therefore, it may be that other potential NLS and intracellular factors alone or together to drive ICP22 into the nucleus. However, this part of the experiment has not been carried out yet, in future, we will study the mechanism of ICP22 incorporation without NLS. In summary, we believe that the transformation of localization from the cytoplasm to the nucleus indicates that the position of synthesis is in the cytoplasm but that the function is exerted in the nucleus.

## Conclusions

The DEV US1 ORF, as IE gene, is 990 bp in length and duplicated within the inverted repeat sequences delineating the US region of the genome. The molecular mass of the ICP22 protein is ~57 kDa, and the protein can enter the nucleus by itself with a classical NLS motif. ICP22 cannot enter the nucleus by itself after mutating amino acid 309R, indicating the amino acid 309R is the key residue. Construction of the US1 mutant viruses and determination of the virus titer indicated that DEV US1 and its NLS are non-essential but associated with a severe growth deficit *in vitro*.

## Data Availability Statement

All datasets generated for this study are included in the article/Supplementary Material.

## Author Contributions

YLi, YW, MW, and AC conceived and designed the experiments. MW, YM, RJ, SC, QY, DZ, ML, and XZ guided the experiment and helped analyse the data. SZ, JH, XO, SM, LZ, YLiu, YY, LP, BT, MR, and XC contributed materials. All authors read and approved the final manuscript.

### Conflict of Interest

The authors declare that the research was conducted in the absence of any commercial or financial relationships that could be construed as a potential conflict of interest.
