# Supplementary figures and images for: Duplicate US1 Genes of Duck Enteritis Virus Encode a Non-essential Immediate Early Protein Localized to the Nucleus
Source: Front Cell Infect Microbiol. 2020 Jan 17;9:463. doi: 10.3389/fcimb.2019.00463 (PMC6979402; doi:10.3389/fcimb.2019.00463)

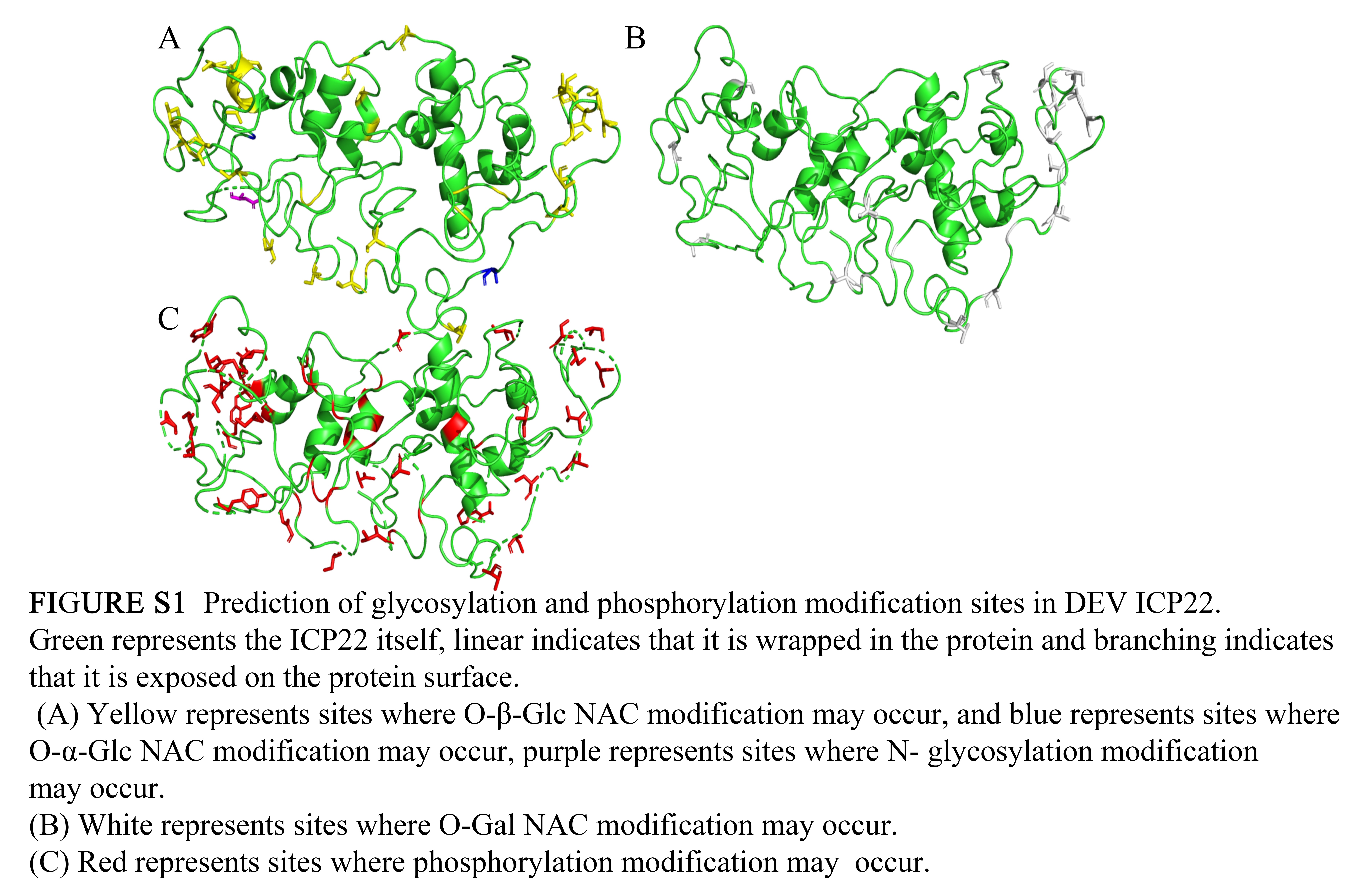

Supplement: Supplementary file 1 [file Image_1.jpg]
